# Evaluating the X-ray-Shielding Performance of Graphene-Oxide-Coated Nanocomposite Fabric

**DOI:** 10.3390/ma15041441

**Published:** 2022-02-15

**Authors:** Serhat Süha Türkaslan, Şule Sultan Ugur, Banu Esencan Türkaslan, Nicholas Fantuzzi

**Affiliations:** 1 Department of Prosthodontics, Faculty of Dentistry, University of Süleyman Demirel, Isparta 32260, Turkey; suhaturkaslan@yahoo.com; 2 Department of Textile Engineering, Faculty of Engineering, University of Süleyman Demirel, Isparta 32260, Turkey; suleugur@sdu.edu.tr; 3 Department of Chemical Engineering, Faculty of Engineering, University of Süleyman Demirel, Isparta 32260, Turkey; banuturkaslan@sdu.edu.tr; 4 Department of Civil, Chemical, Environmental and Materials Engineering, University of Bologna, 40126 Bologna, Italy

**Keywords:** X-ray shielding, graphene oxide, layer by layer, environmentally friendly, nanocomposite

## Abstract

Exposure to ionizing radiation (IR) during diagnostic medical procedures brings certain risks, especially when experiencing recurrent exposures. The fabrication of nano-based composites, doped with different nanoparticles, have been suggested as effective shielding materials to replace conventional lead-based ones in material sciences and nanotechnology. In this study, commercially available fabrics, used to produce scrubs and gowns for clinical staff, are modified utilizing graphene oxide (GO) nanoparticles using a layer-by-layer (LBL) technique. GO was obtained from graphite through environmentally friendly technology by using a modified–improved Hummers’ method without NaNO_3_. Lightweight, flexible, air- and water-permeable shielding materials are produced that are wearable in all-day clinical practice. The nanoparticles are kept to a minimum at 1 wt%; however, utilizing the LBL technique they are distributed evenly along the fibers of the fabrics to achieve as much shielding effect as possible. The evaluation of samples is accomplished by simulating real-time routine clinical procedures and the radiographic programs and devices used daily. The GO-coated nanocomposite fabrics demonstrated promising results for X-ray shielding.

## 1. Introduction

Radiology has significant diagnostic benefits and is mandatory for a precise treatment plan, but exposure to ionizing radiation (IR) brings certain risks [1]. Both the National Radiation Protection Board (NRPB) and the International Commission on Radiation Protection (ICRP) confirm that there is no “safe” dose for radiation, and that any tissue exposed has the potential to induce malignant changes [2].

In dentistry, imaging doses for diagnostic purposes may be considered as low, when compared with treatment levels [3]. However, with the impact of recurrent exposures to dental X-ray radiation and prevalence, even a small increase in thyroid cancer risk would be a noteworthy concern for public health [4].

A survey reports that the majority of dentists use different radiation settings, suggesting lower doses for children than for adults, but 30% of dentists evaluated in this study still use the same setting both for adults and children. Although rectangular collimators are reported to reduce patient IR exposure by 60–80 percent compared with circular collimators, 99.4% of dentists surveyed in 2014–2015 continue to use circular collimation, leading to scatters [5].

From another point of view, it is clear that shielding radiosensitive organs, such as gonads, eye lenses, breasts or thyroid glands, must be achieved against scatters [6]. Additionally, it is a well-known fact that pediatric patients are extremely radiosensitive, they have increased mitotic activity and longer life expectancy compared with adults and consequently a greater possibility for radiation-induced cancer [7].

Approximately 6–21% of patients exposed to dental radiographs are under 15 years of age due to various reasons, such as orthodontics or trauma. Although 2D imaging has been applied traditionally, in recent years, increasing use of cone beam computed tomography (CBCT) is reported, especially for orthodontic treatment, and there is no diagnostic reference level (DRL) found in the contemporary literature for CBCT [8].

It is also reported that IR may not only be the cause of radiation-induced cancer, but low-dose IR may also cause detrimental effects in the brain or neurons, engendering and provoking elevated reactive oxygen species levels, leading to oxidative stress, mitochondrial dysfunction, loss of synaptic plasticity, alterations in neuronal structure and impaired microvascular changes.

These changes may cause neurodegeneration, causing dementia, multiple sclerosis, etc., in the elderly age group. Shielding and protective equipment remains an issue, not only for children and radiosensitive organs [9], but also for tissues which undergo a very low level of cell division and are thought to be robustly resistant to radiation effects [5].

Although the use of wearable products for shielding against X-ray irradiation is legally compulsory in medical fields, products used for this requirement are a modified form of non-wearable materials. Lead plates are generally sewn to a polymer with the intention of creating a wearable form. Nevertheless, the heavy weight, toxicity, detachment and fracture problems caused by these have been the most important focus of research in the recent literature [10,11,12]. It is hard and/or time consuming to protect different organs with several items of lead shielding equipment, and this becomes heavier for children especially. In addition, working long hours with heavy equipment is inconvenient for technicians. Inhalation is another risk factor, as floating microparticles in the air has been reported [13,14]. Lead exposure may also induce several biological complications depending on the level and the duration of exposure. Several studies have demonstrated the toxic effects of lead. The immune system may be negatively affected, increasing allergic reactions, infectious and autoimmune diseases or malign neoplasms [15]. Additionally, the reproductive systems of both male and female patients are affected by repeated, high-dose exposures to lead. Among all tissues, the brain is the most sensitive organ to lead exposure. Children are demonstrably more sensitive than adults; lead may affect synapse formation in the cerebral cortex, also interfering with the development of neurochemicals, including neurotransmitters, and the organization of ion channels [16,17].

These shortcomings encourage researchers to seek alternatives to lead material for shielding, or to develop ecofriendly composites [18]. In general, non-lead shielding materials are fabricated with additives and binders mixed with attenuating heavy metals. A few manuscripts have been published about radiopaque particles during the fiber production process [19,20]. Numerous studies focus on developing contemporary fabrics for X-ray shielding, generally utilizing conventional coating methods and composites doped with different metal nanoparticles to replace conventional lead-based materials. Several nanomaterials, such as tungsten, bismuth, phthalonitrile, molybdenum, carbon allotropes (graphene, graphene oxide, etc.) and their composites, have been used as an effective radiation-shielding material [14,21,22,23].

The aim of this study was to develop a lightweight, user-friendly, wearable shielding material. Commercially available fabrics which are used to produce scrubs and gowns for clinical staff were modified utilizing graphene oxide (GO) nanoparticles, using a layer-by-layer (LBL) technique. Our goal was to achieve air and water permeability of the fabric, alongside low film thickness and constant abrasion stability to prevent the material from becoming stiffer. Therefore, the fibers of the fabric were coated with 1 wt% GO nanoparticles with the LBL technique, and the shielding performance was evaluated by a simulation of a real-time clinical procedure. The X-ray attenuation characteristics of novel GO-based nanocomposite fabrics for shielding, used in low-energy diagnostic applications, is presented.

## 2. Materials and Methods

### 2.1. Graphene Oxide (GO) Synthesis

GO was synthesized with a new methodology we have developed called modified–improved Hummers. Briefly, 2 g flake graphite (99% carbon) and H_2_SO_4_ were mixed in an ice water bath and 6 g KMnO_4_ was gradually added and stirred for 2 h. When the mixture became pasty, 300 mL deionized water (DI) was added, and the temperature was raised to 90 °C. Then, H_2_O_2_ and HCl were added to the mixture, respectively, and centrifuged to remove unexfoliated graphite.

### 2.2. Nanocomposite Fabric Preparation Method

The LBL technique is based on replacing oppositely charged polyelectrolytes by electrostatic attraction. This method aims to achieve the easy preparation of nanocomposite textile fibers by enabling the production of functional textile materials for protective clothing. The properties of simplicity, operability, universality and thickness, controlled at the nanoscale level, make the LBL assembly technique superior to other traditional coating methods [24,25].

Mercerized and bleached 100% cotton woven fabric (plain weave, 284 g/m^2^, 20 ends/cm, 40 picks/cm) and 100% polyester fabric (plain weave, 162 g/m^2^, 22 ends/cm, 65 picks/cm) were used as substrate for the LBL process.

**Figure 1:**
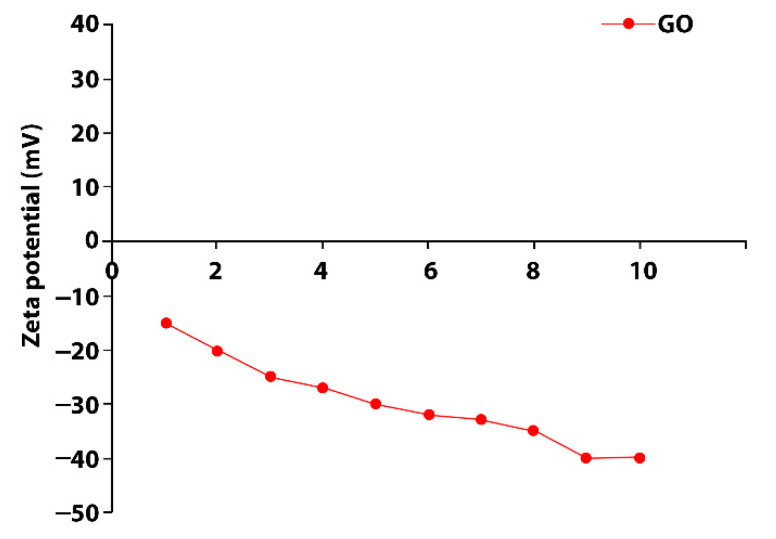
Zeta potential graphic of GO [26].

Before multilayer film coating process, fabric surfaces were pretreated with polyethylenimine (PEI, 0.1 g/L, PH: 10, dip-coating method) to obtain cationic surface charges. GO suspension was prepared at 50 watt for 2 h by an Ultrasonic Homogenizer (Vibra-Cell, Sonics, Rome, Italy). The concentration of suspension was adjusted to 0.1 wt%. According to the ASTM D 4187-82 standard, zeta potential values between 30 and 40 mV/−30 and −40 mV represent moderate stability. These values indicated that all coated fabrics remained stable in water. Zeta potential can be an index to the stability of the GO suspensions. Figure 1 shows that negative zeta potential occurred with the GO suspensions at pH 5.5 between −30 and −40 mV by using HCL (Figure 1).

A laboratory-type padding machine was used for the LBL deposition process. In the deposition process, the positively charged polyester/cotton fabrics were padded with the following solutions, alternately: (a) anionic GO polyelectrolyte solution; (b) deionized water; (c) cationic poly(diallyldimethylammonium chloride) (PDDA) solution; (d) deionized water. This deposition cycle was repeated until 20, 30 and 40 multilayer GO/PDDA films were deposited on the fibers. Multilayer-film-coated fabrics were dried at 80 °C for 10 min using a laboratory-type furnace, then the temperature was increased to 105 °C and fabrics were cured at this temperature for 5 min.

### 2.3. X-ray Shielding Simulation of Nanocomposite Fabrics

The shielding performance of the GO-coated cotton and polyester fabrics against X-ray beams were evaluated using radiovisiography (Romexis, Planmeca, Helsinki, Finland). The experiment dose was 0.56 s irradiation with at 60 kV, 5 mA energy, equivalent to a single periapical dental radiograph dose—the recommended setup for adults by the manufacturer—in order to simulate routine daily practice. Single or double (2-folded) cotton and polyester fabrics were evaluated in 4 subgroups as untreated (UT), 20-layer (20L), 30-layer (30L) and 40-layer (40L) fabrics.

### 2.4. Experimental Setup

**Figure 2:**
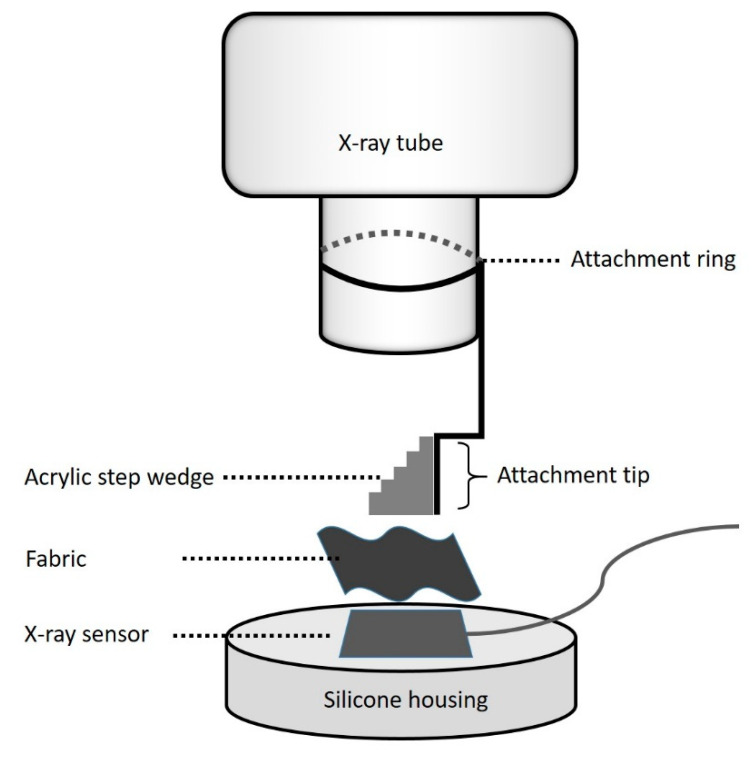
Schematic demonstration of the setup.

To obtain reproducible and standardized images, a setup was constructed for the samples based on the specimen used. The base was the holder for the X-ray sensor which was fabricated utilizing C-type silicone impression material with accurate recess for the X-ray sensor. The upper element was the step-wedge, which was attached perpendicular to one end of the plastic holder, which was located at the tip of the tube using a precise attachment ring. The top and the base part of the assembly remained intact during the test period but the tested fabric materials in between were changed. Polyester and cotton samples with varying GO layers consisting of a single fabric or double (2-folded) fabric were positioned under acrylic step-wedge consecutively and the digital images were recorded for evaluation (Figure 2).

### 2.5. Characterization Nanocomposite Fabrics

The GO characterization was performed with X-ray diffraction (XRD) and scanning electron microscopy (SEM/EDX) technique. In order to verify the multilayer coat of GO on the fabrics, the samples were also evaluated using a scanning electron microscope (SEM, Quanta Feg 250; FEI, Eindhoven, the Netherlands). GO and GO-coated multilayer composites were examined with a low vacuum at 20.00 kV and 12.7–13.2 mm working distance, and at 10,000× and 3000× magnifications, respectively. Elemental analysis of GO was carried out using SEM microscope equipped with an energy-dispersive X-ray spectroscopy (EDX, Quanta Feg 250; FEI, Eindhoven, The Netherlands). The distribution and atomic composition of GO was examined using elemental mapping at an accelerating voltage of 20 kV. The crystalline phase and size of GO were examined by X-ray diffraction (XRD, Bruker D8 Advance Twin-Twin; Bruker, Karlsruhe, Germany). GO was examined at 40 kV, 40 mA, and 1600 watts. Data acquisition was performed using scan speed 2°/min, at a sampling width of 0.01° from 5° to 70° (2θ).

### 2.6. Assessment of The Data

Digital images were obtained when the acrylic step-wedge and test samples were subjected to the X-ray. A line profile tool in the digital program (Romexis, Planmeca, Helsinki, Finland) that evaluates the value of the image color in terms of grayscale was used. The data were listed, and a diagram was formed according to the results. The software package SPSS-25.0 was used to perform statistical data analysis. One-way ANOVA was performed to compare UT, 20L, 30L and 40L variable scores according to different step-wedge thickness measurements, and a Bonferroni multiple comparison test was performed for all pairwise differences between the means. A *p* value ≤ 0.05 was considered a statistically significant result.

## 3. Results

**Figure 3:**
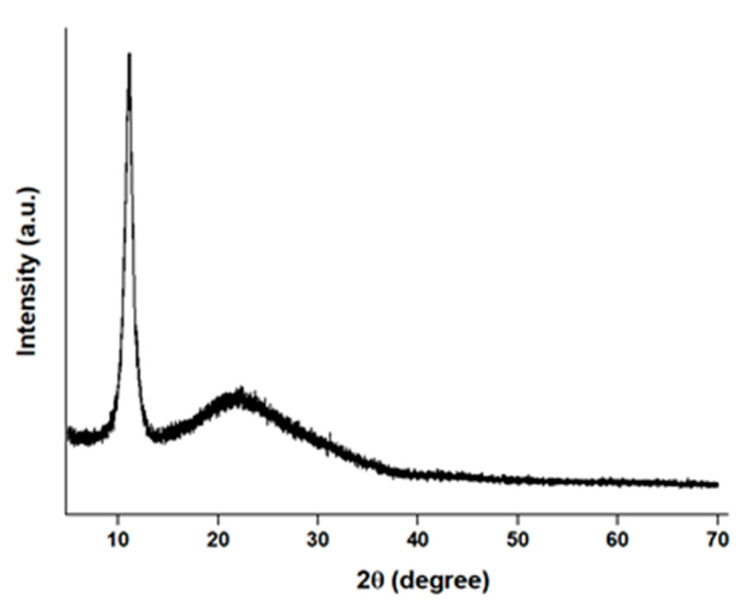
XRD patterns of GO.

The characteristic peak of GO located at about 2θ = 11.2 nm indicates that the product was oxidized, and that the exfoliation process increased the d-spacing to 0.81 nm (Figure 3) [27].

The 17 layers of GO were obtained from 47-layer flake graphite, and this data was calculated using Scherrer equation. Crystal size (11.93 nm) and interlayer distance (0.81 nm) were revealed from XRD spectroscopy.

**Figure 4:**
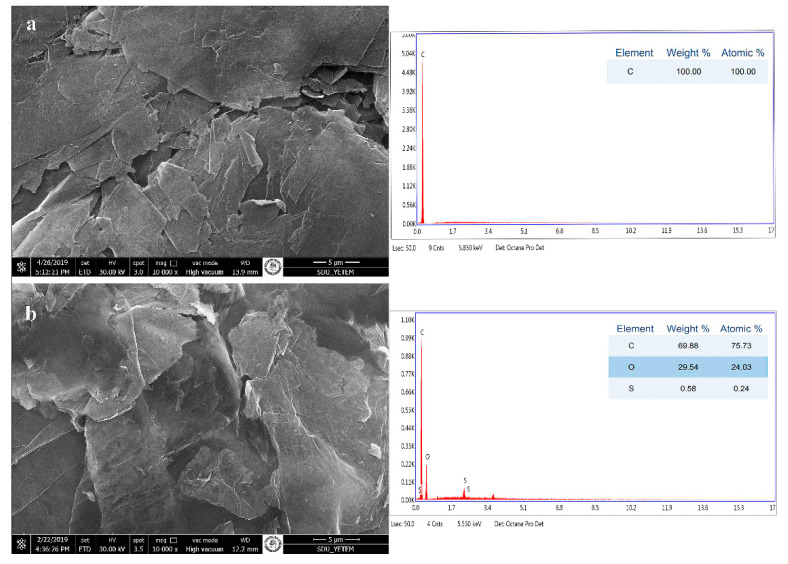
SEM/EDX image of graphite (**a**) and GO (**b**).

As seen in EDX analyses, functional groups were added between the layers as a result of oxidation of the graphite forming the layered GO morphology (Figure 4).

**Figure 5:**
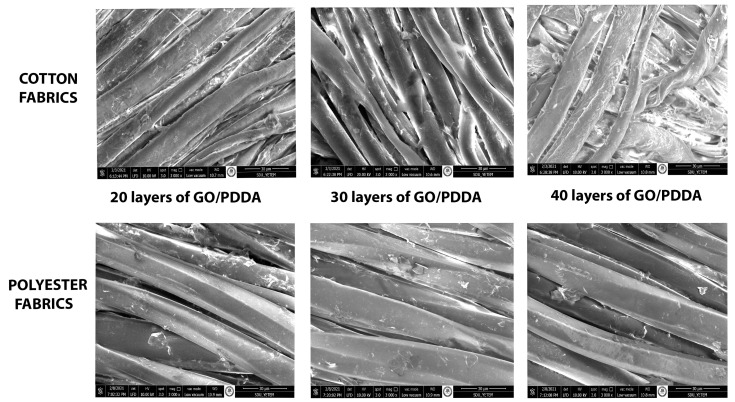
SEM micrographs of GO-multilayer-coated fabrics.

GO-multilayer-coated fabrics SEM micrographs are shown in Figure 5. GO particles could be seen on the fiber surfaces and, as layer numbers increase, GO particle density has increased as expected. In multilayered films, due to cationic PDDA suspension being deposited on the top of the film, GO particles were not clearly observed in the SEM micrographs in the graphene plates.

**Figure 6:**
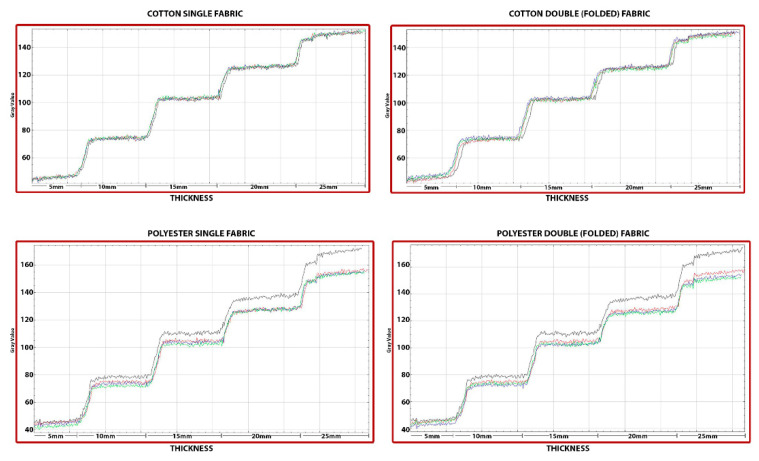
The gray scale values of GO multilayer coated fabrics. Black (UT), red (20L), blue (30L) and green (40L) lines indicate the differences.

Figure 6 demonstrates decrease in the optical density by increasing the thickness of the step-wedge. Additionally, the increase in the number of the GO layers that coats the fibers of the fabric by means of LBL technique leads to a decrease in optical density, which, in turn, means a better shielding potential. A hundred numeric data, obtained by the use of a digital greyscale, were evaluated for each level of thickness (Figure 6).

According to the analysis result, no statistically significant difference was found for the single cotton fabric sample (*p* > 0.05) between UT, 20L, 30L and 40L measurements on 5 mm and 10 mm thickness measurement groups. However, the results from the ANOVA test indicated a statistically significant difference between UT, 20L, 30L and 40L measurements on the 15 mm (F = 9.051, *p* = 0.000), 20 mm (F = 9.212, *p* = 0.000) 25 mm (F = 29.864, *p* = 0.000) thickness groups.

Regarding the double cotton fabric sample, results from the ANOVA test indicated a statistically significant difference between UT, 20L, 30L and 40L measurements on the groups which are 5 mm (F = 62.704, *p* = 0.000), 10 mm (F = 29.759, *p* = 0.000), 15 mm (F = 25.484, *p* = 0.000), 20 mm (F = 35.628, *p* = 0.000) and 25 mm (F = 57.290, *p* = 0.000) thickness.

When evaluating polyester fabrics, the results of both single and double samples were significantly different for all thicknesses. The results from the ANOVA test indicated a statistically significant difference between UT, 20L, 30L and 40L measurements on all the thickness groups.

## 4. Discussion

It is not possible to formulate a realistic approach to comparing the risks of dental IR with background radiation, which is present in the environment—not a product of deliberate introduction of radiation sources. The repeated doses of dental X-rays directed to the head area, containing the brain and neighboring thyroid gland, cannot be compared to an equal amount of background IR [5].

In order to estimate the variation of the dosages, a full-mouth series of 18 intraoral images obtained using digital or E- and F-speed film and rectangular collimation is assumed to be equal to 4.3 days of background radiation [28].

A comparison could be reasonable by using the radiation unit Sievert (Sv). A single intraoral tooth X-ray delivers an average dose of 0.8 μSv [29]. Panoramic X-rays may provide one–five times higher doses than a full-mouth series, depending on the brand and the model. When evaluating CBCT radiology, a metanalysis comparing different CBCT brands and models found that adult exposure ranged from 5 to 1073 μSv. Child doses ranged from 7 to 769 µSv [30]. Therefore, it can be estimated that a single dental appointment may cause varying doses from 0.8 μSv up to 1073 μSv for a patient, and repeated doses for the clinical staff for each patient.

This study demonstrates a feasible shielding material that enabled the transformation of routine, medically used fabrics into X-ray-shielding materials, while avoiding hindering their physical properties, such as flexibility, light weight and breathability.

Studies usually evaluate the shielding potential of various materials via simulation programs, i.e., [31,32,33] in order to save time or due to their financial advantages. In this study, we preferred to evaluate the samples by simulating real-time routine clinical procedures and the radiographic programs and devices used daily. The acrylic step-wedge helped to simulate differing distances. In this way, the shielding potential of the samples were evaluated with the same conditions of routine clinical cases.

Various studies have evaluated the shielding potential of single- and multilayered shields composed of barium, bismuth, gadolinium, tin and tungsten, with varying filler ratios from 8 to 50 wt%. Most studies are simulations using Monte Carlo and few are experiments developing environmentally friendly shields, which are lighter than conventional lead aprons, but increasing the filler ratio may adversely affect the physical properties, such as flexibility or permeability. The GO–filler ratio was kept constant and at a minimum with 1 wt% in this study in order to keep the changes in physical properties of the samples, such as the light weight, flexibility or breathability, at a minimum level [34,35,36,37,38,39].

It is important to note that the nanostructured materials can be produced and coated or painted practically, to conform to any shape of interest [22,40,41,42]. Therefore, they can be applied when producing different radiation protection equipment, such as thyroid shields and protective aprons. Additionally, some equipment that is not specially fabricated for shielding, but for daily routine use for protection against cross-contamination, such as bonnets or gloves, can also be coated. This will render possible a dual benefit of these materials. With the advancements in nanotechnology, the current trend is towards exploiting the properties of nanostructured material in order to create advanced nanocomposites for effective, lightweight, durable, radiation-resistant equipment [40,41,42].

According to different studies, at lower energy levels, composites containing nanoparticles reduce X-rays better than micro particles. This can be clarified by more uniform dispersion of nano-sized materials, that interact an X-ray photon with lower energy. The X-ray photon is absorbed more in nanoparticle-coated composites than other materials, such as micro-coated materials or dense objects. In an equal mass of nano- and microstructured materials, the number of nanoparticles is higher than microparticles, with a higher surface-to-volume ratio. In other words, nanostructured composites consist of a higher number of particles per gram when compared with microstructured composites. Therefore, GO in nanoform is coated on fibers of two different fabrics and LBL technique is used to distribute the nanoparticles on fibers of the fabric more evenly for better shielding effect. Thus, nanostructured shielding materials can be lighter than microstructured ones, as well as providing equal radiation attenuation [38,43].

The statistical analysis revealed that no difference was observed between NT and all layers with 5 or 10 mm thickness when single cotton fabric was evaluated. On the contrary, polyester fabric demonstrated statistically different results. This may be related with the compactness of the fibers in the fabrics, or fiber thickness may affect the shielding potential. One of the limitations of the present study was that the experiments were performed using only two different types of fabrics. Fibers different than cotton and polyester or hybrid ones may present different results. Another issue is the orientation of the fibers. Fabrics produced with the same material but with different fiber orientations, in other words, different knitting processes, have not been evaluated in this study.

The results obtained when evaluating cotton samples demonstrates no statistically significant data with relatively thin objects. However, when the thickness of the step-wedge increased, significant data was obtained. The results show that the shielding potential of GO-coated fabric increases by the thickness of the step-wedge that mimics the distance. The staff in the clinic are not always subjected to X-ray at close distances. This wearable product as a lighter alternative to lead aprons and may be suitable for routine use, ensuring protection during all-day practice, especially for scatters and long-distance exposures.

## 5. Conclusions

The current study indicates that the use of nanotechnology offers new possibilities in the production of radiation-shielding materials that are customizable. The incorporation of GO as nanomaterial within fibers of different fabrics allows the production of shielding materials that could replace lead as the dominating material in radiation shielding.

In summary, this study presents the development of an X-ray-shielding, nano-GO-composite-coated material. The material is produced without hindering the physical properties, such as air and water permeability, light weight, and flexibility, which are valuable in daily use of clinical textiles. Nano-coated, GO-composite fabrics ensure a feasible and trustworthy shielding material when compared with lead aprons. Further investigation must be considered clinically for optimum results.

## Data Availability

The processed data required to reproduce these findings is available from the authors upon reasonable request.
